# Characterization of tetracycline modifying enzymes using a sensitive *in vivo *reporter system

**DOI:** 10.1186/1471-2091-11-34

**Published:** 2010-09-11

**Authors:** Zhou Yu, Sean E Reichheld, Leslie Cuthbertson, Justin R Nodwell, Alan R Davidson

**Affiliations:** 1 Department of Molecular Genetics, University of Toronto, 1 King's College Circle, Toronto, ON, M5S 1A8, Canada; 2 Department of Biochemistry, University of Toronto, 1 King's College Circle, Toronto, ON, M5S 1A8, Canada; 3 DeGroote Institute for Infectious Diseases Research, Department of Biochemistry and Biomedical Sciences, McMaster University, 1200 Main Street W, Hamilton, ON, L8N 3Z5, Canada

## Abstract

**Background:**

Increasing our understanding of antibiotic resistance mechanisms is critical. To enable progress in this area, methods to rapidly identify and characterize antibiotic resistance conferring enzymes are required.

**Results:**

We have constructed a sensitive reporter system in *Escherichia coli *that can be used to detect and characterize the activity of enzymes that act upon the antibiotic, tetracycline and its derivatives. In this system, expression of the *lux *operon is regulated by the tetracycline repressor, TetR, which is expressed from the same plasmid under the control of an arabinose-inducible promoter. Addition of very low concentrations of tetracycline derivatives, well below growth inhibitory concentrations, resulted in luminescence production as a result of expression of the *lux *genes carried by the reporter plasmid. Introduction of another plasmid into this system expressing TetX, a tetracycline-inactivating enzyme, caused a marked loss in luminescence due to enzyme-mediated reduction in the intracellular Tc concentration. Data generated for the TetX enzyme using the reporter system could be effectively fit with the known *K*_m _and *k*_cat _values, demonstrating the usefulness of this system for quantitative analyses.

**Conclusion:**

Since members of the TetR family of repressors regulate enzymes and pumps acting upon almost every known antibiotic and a wide range of other small molecules, reporter systems with the same design as presented here, but employing heterologous TetR-related proteins, could be developed to measure enzymatic activities against a wide range of antibiotics and other compounds. Thus, the assay described here has far-reaching applicability and could be adapted for high-throughput applications.

## Background

Over many decades, a wide variety of *in vitro *and *in vivo *screens have been used to identify small molecules with useful activities, such as antibiotics and enzyme inhibitors. However, there is still a need for simple and widely applicable assay systems for characterizing the activity of enzymes against specific small molecules that avoid the necessity of enzyme purification or high level expression. In the work described here, we have developed an *in vivo *luminescence-based reporter system that can be used to detect and characterize enzymatic activities against the antibiotic, Tetracycline (Tc). In the future, systems designed on the same principle could be used to investigate enzymes active against a variety of other small molecules.

Tc and its derivatives are highly effective broad specificity antibiotics that have been widely used for many decades [1]. The ubiquitous utilization of tetracyclines has resulted in the emergence of numerous resistance mechanisms mediated by a variety of proteins including efflux pumps, drug modifying enzymes, and ribosome protection factors [2]. The significant negative clinical impact of resistance to tetracyclines has led to intensive efforts to elucidate the mechanisms of this resistance and to develop new Tc derivatives that will overcome resistance mechanisms. To this end, much research has focused on enzymes capable of modifying tetracycline derivatives, either as a resistance mechanism or as a step in the tetracycline synthesis process [3]. It is hoped that characterization of these enzymes will lead to approaches for combating resistance and creating more potent tetracycline derivatives. Although *in vitro *spectroscopic methods are available to assess some enzymatic modifications of tetracyclines, *in vivo *assays of these enzymes are quite complicated if the enzyme activity does not confer resistance to the growth inhibitory effect of the antibiotic [4]. To aid in characterizing enzymes that modify tetracyclines and in identifying novel enzymes active against tetracyclines, a simple *in vivo *method to detect these activities would be very useful.

**Figure 1:**
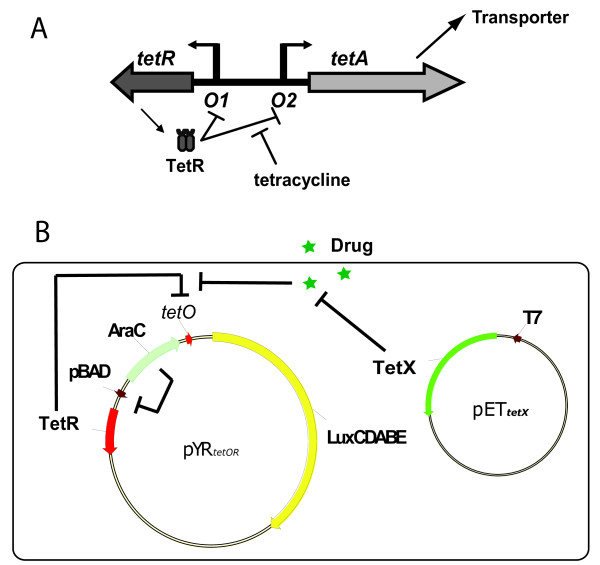
**The TetR regulon and pYR cell based repression-induction system**. **A) **The tetracycline resistance conferring *tetR-tetA *regulon. The repressor gene, *tetR*, is depicted as a dark gray arrow and the resistance gene, *tetA*, as a light gray arrow. TetR regulates the transcription of *tetA *and its own gene by binding operators O1 and O2. The binding of TetR to the operator sequences is inhibited by interaction with Tc. **B) **Plasmid map of pYR_*tetOR *_and the scheme of the cell based repression-induction system. TetR expressed from the *pBAD *promoter on pYR_*tetOR *_represses trancription of the *lux *genes on the same plasmid. Upon addition of Tc, TetR is induced, the *lux *genes are transcribed, and luminescence is emitted from the cells. If active TetX enzyme is produced from pET_*TetX*_, then the effective concentration of Tc is reduced by the activity of the enzyme and the transcription level of the *lux *genes is also reduced, resulting in decreased luminescence. The box represents the *E. coli *cell membrane.

In the work presented here, we describe a system for characterizing tetracycline-modifying enzymes that takes advantage of the tetracycline repressor (TetR). Many of the genes conferring resistance to tetracyclines are regulated by TetR, which was first isolated and characterized more that 25 years ago [5]. TetR is homodimeric with each monomer composed of an N-terminal DNA-binding domain, and a C-terminal domain that mediates dimerization and binds to tetracyclines [6]. In the typical TetR-regulated regulon, TetR binds to two DNA operator sites, thereby repressing transcription of its own gene as well as the divergently transcribed *tetA *gene, which encodes an exporter of tetracyclines (Figure 1A). Binding of tetracyclines to the C-terminal domain of TetR leads to a conformational change in the DNA-binding domains, which causes them to lose affinity for DNA, relieving repression of the *tetR *and *tetA *genes. TetR is the founding member of a huge family of transcriptional regulators, which we refer to as the TetR family of transcriptional regulators (TFRs). TFRs constitute the third most frequently occurring transcriptional regulator family found in bacteria [7] with more than 10,000 proteins in the non-redundant protein database annotated as members of this group. TFRs have been identified that control the expression of genes conferring resistance to most known antibiotics including tetracycline, chloramphenicol, erythromycin, ampicillin, and streptomycin [6]. As well, TFRs are involved in the regulation of many aspects of bacterial physiology in response to various small molecule inducers, including quorum sensing, biofilm formation, morphological differentiation and antibiotic production. All characterized TFRs are homodimers with the same domain structure and general fold as TetR, and most of them function in a manner similar to TetR, mediating transcriptional repression that is relieved only in the presence of their specific small molecule ligand. TFR sequences in bacterial genomes can be reliably identified due to the high level of sequence conservation in their N-terminal DNA-binding domains [6]; however, their ligand-binding domains display tremendous diversity commensurate with the broad range of ligands recognized by TFRs.

In a previous study, we designed a TetR-based biosensor that produced luminescence upon addition of tetracycline derivatives [8]. The goals of the work described here were to improve the sensitivity of this system and to then exploit it to detect the activities of tetracycline modifying enzymes. To this end, a TetR regulated transcriptional promoter was cloned upstream of the *lux *operon in such a way that luminescence was elicited at very low concentrations of Tc. We then demonstrated that introduction of the Tc-modifying enzyme, TetX [9], into this system led to a significant reduction in luminescence due to the activity of the enzyme, which degrades the inducer of TetR. This reporter system could be modified to both identify ligands for TFRs of unknown function, and to detect enzymes active against these ligands. Since there are currently at least 50 known ligands for TFRs [6], the assay principle described here is applicable to the characterization of a considerable number enzymes active against small molecules.

## Results and Discussion

### Construction of a Sensitive Luminescence-Based System for the Detection of Inducing Ligands for Members of the TetR family of Repressors

**Figure 2:**
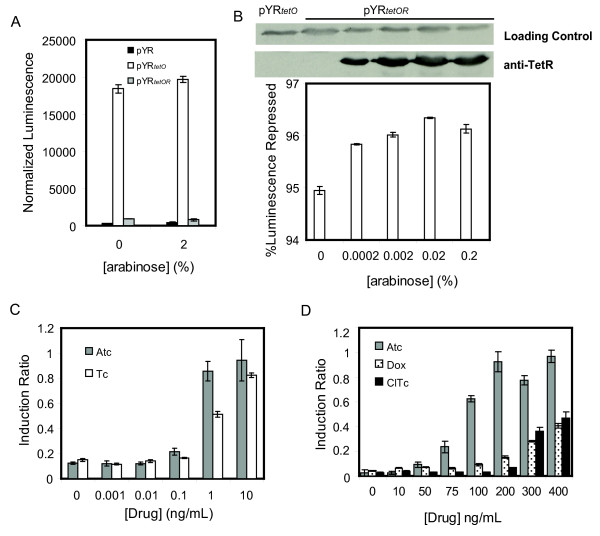
**Luminescence production by the pYR plasmids in the presence or absence of Tc derivatives**. **A) **Luminescence produced by pYR, pYR_*tetO *_and pYR_*tetOR *_harboring cells. **B) **Degree of repression by TetR at various arabinose concentrations. TetR expression levels are indicated by an anti-TetR western blot on top of the plot. **C) **Induction of TetR in pYR_*tetOR *_bearing cells at different concentrations of Tc and Atc in the absence of arabinose. **D) **TetR induction in pYR_*tetOR *_bearing cells induced by different concentrations of Atc, Dox and ClTc in 0.02% arabinose.

We previously constructed a *lux*-based biosensor system to investigate the binding of TetR to its DNA binding site (*tetO*), and to measure transcriptional induction elicited by tetracycline (Tc) and its derivatives [8,10]. For the work described here, we sought to create a more sensitive Tc-responsive system. We placed the *luxCDABE *gene cluster under the control of a TetR-repressible promoter on a low copy number pSC101-derived plasmid [11]. This plasmid, named pYR_*tetO*_, mediated production of a high level of luminescence in *E. coli *(Figure 2A, white bars), while a plasmid containing the *lux *genes with no promoter (pYR) produced no detectable luminescence (Figure 2A black bars). TetR was then cloned into the same plasmid under the control of the arabinose-inducible pBAD promoter to produce pYR_*tetOR *_(Figure 1B). Expression of TetR in pYR_*tetOR *_led to repression of luminescence (Figure 2A, gray bars) caused by the binding of TetR to the *tetO *site in the promoter of the *lux *genes. Repression occurred even in the absence of arabinose, indicating that the small amount of TetR expression from the pBAD promoter in the absence of arabinose was sufficient for repression of *lux *expression, even though TetR expression could not be detected by western blot under these conditions. Addition of arabinose only led to a small reduction in luminescence (Figure 2B).

To demonstrate the utility of our system for detecting Tc and its derivatives, luminescence production from pYR_*tetOR *_was measured in the presence of varying concentrations of these antibiotics. In these experiments the degree of induction is expressed as an induction ratio, the luminescence generated from pYR_*tetOR *_divided by that from pYR_*tetO *_(Figure 2A, white bars). Significant luminescence production was observed at Tc concentrations as low as 1 ng/mL, which is far below its minimum inhibitory concentration (MIC) in *E. coli *of 500 ng/mL (Figure 2C, white bars) [12]. Anhydrotetracycline (Atc), a stronger inducer of TetR [13], also displayed a greater ability to induce TetR in this assay, relieving repression of the *lux *operon at a concentration of only 0.1 ng/mL. The addition of 0.02% arabinose to the system, which greatly increases the intracellular concentration of TetR (Figure 2B), led to a requirement for much higher concentrations of Tc and Atc, as well as the Tc analogs doxycycline (Dox) and chlorotetracycline (ClTc), to relieve *lux *repression (Figure 2D). For example, full induction with Atc under these conditions required a concentration of 200 ng/mL, indicating that minimizing the cellular concentration of TetR increases the sensitivity of the reporter system. Concentrations of Tc high enough to induce luminescence under conditions of increased TetR expression caused significant inhibition of cell growth (data not shown). Together, these data demonstrate that the pYR_*tetOR *_system can detect very low concentrations of Tc and its derivatives. The reporter system is sensitive to the concentration and chemical properties of inducer molecules, and to the intracellular concentration of TetR.

### The Detection of Tc-Modifying Enzymatic Activity In Vivo Using the pYR_tetOR _Luminescence System

**Figure 3:**
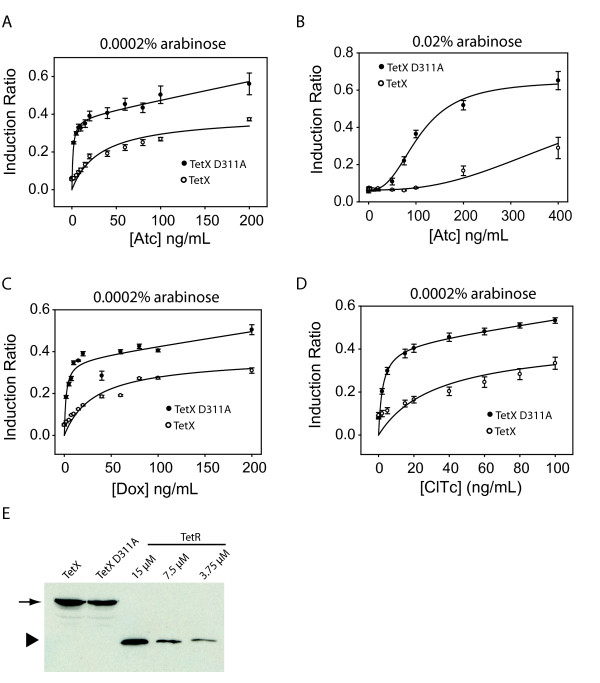
**Activities of TetX detected using the pYR**_***tetOR ***_**system and fluorescence-based assays**. **A-D) **Induction ratios in the presence of the active enzymes (open circles) or mutated inactive enzymes (solid circles). Best fit lines using the enzyme kinetics model (see text and Methods for details) are shown. A, C and D were performed in the presence of 0.0002% arabinose, which was the minimum arabinose concentration where consistent full repression could be obtained under these conditions. B was perfomed in 0.02% arabinose. **E) **A Western blot (anti-6xHis) showing the expression level of TetX at the point where drugs were added. Bands indicated by arrows correspond to the enzymes and bands indicated by the triangle correspond to purified TetR-6xHis protein. The enzyme bands show the total amount of enzyme from ~1 μL cell pellet, and the TetR bands show the amount of 6xHis tagged protein from 1 μL solution containing the indicated concentration protein.

To test the ability of the pYR_*tetOR *_system for *in vivo *detection of enzymatic activity against Tc, we investigated the TetX enzyme. TetX is an FAD-dependent monooxygenase from *Bacteroides fragilis *that has been shown to hydroxylate Tc creating an unstable compound that undergoes rapid decomposition [9]. To measure the effect of TetX when expressed in pYR_*tetOR*_-containing cells, we introduced a separate plasmid into these cells that expressed this enzyme (pET_*tetX*_, Figure 1B). For comparison, we also tested pYR_*tetOR*_-containing cells co-transformed with a plasmid expressing the D311A mutant of TetX (pET_*tetXD*_), which is substituted at a highly conserved residue in the FAD-binding site and was expected to possess no enzymatic activity (Additional file 1: Figure S1). We verified that the pET_*tetX *_construct produced active TetX and that the D311A mutant was reduced in activity using an *in vitro *fluorescence based assay for TetX activity (Additional file1: Figure S2). As shown in Figure 3A, we measured the luminescence generated by pET_*tetX*_- and pET_*tetXD*_-containing cells at varying concentrations of Atc, and found that considerably less light was emitted by cells containing the plasmid expressing the WT version of TetX. For example, pET_*tetX*_-bearing cells required a concentration of ~100 ng/mL Atc to generate a level of luminescence similar to that emitted from pET_*tetXD*_-containing cells at an Atc concentration of only 2 ng/mL. We surmised that the reduction in luminescence in pET_*tetX*_-containing cells was the result of TetX-mediated catalysis of Atc into an unstable product and/or a product that could no longer bind TetR. Thus, the concentration of Atc available within the cells to bind TetR was decreased, which led to a greater degree of transcriptional repression of the *lux *genes by TetR (Figure 1B). We found that a similarly large reduction of luminescence was elicited by pET_*tetX *_when the assay was done under conditions of high TetR expression (0.02% arabinose) even though induction did not occur until a much higher concentration of Atc was added (Figure 3B). Assays performed with Dox and ClTc indicated that, as expected [9], TetX was also active against these Tc derivatives since reduced luminescence was observed in pET_*tetX*_-containing cells treated with these compounds (Figures 3C, D). It should be noted that the expression levels of WT TetX and TetX-D311A were similar (Figure 3E), indicating that the luminescence differences observed above were due to differences in the activity of these enzymes. Similar reductions in luminescence were observed when cells were treated with Tc (data not shown).

### Quantitative Analysis of the In Vivo TetX Assay

Our success in detecting the enzymatic activity of TetX in a cell based assay prompted us to determine whether the behavior observed in our assays could be accounted for by the known kinetic parameters of the TetX enzyme. To this end we formulated a series of equations to describe the TetX enzymatic activity within the cell based system in terms that were as simple as possible (see Methods for details). The objective of our analysis was to account for the difference between the dose-response curves generated in the presence of TetX as compared to TetX_D311A_, which we showed above is an inactive enzyme. Our equations were predicated on the assumption that once tetracycline is added, the media becomes an infinite drug reservoir. In the absence of TetX, drug molecules enter cells from the media driven by diffusion and rapidly reach an effective steady state concentration, which is referred to as [*I*_*eff*_]. With TetX present inside the cell, the intracellular concentration of drug is simultaneously increased by the process of diffusion and decreased by the enzymatic activity of TetX. The intracellular drug concentration reaches equilibrium only when the rate of inward diffusion is matched by the rate of enzymatic modification. Thus, the final effective concentration of drug within these cells ([*I*_*eff*_]) is a function not only of the extracellular concentration of drug ([*I*_*out*_]), but also the rate of drug diffusion, the enzymatic activity of TetX, and the time taken after drug addition for the intracellular drug concentration to reach equilibrium (*t*).

In our data fitting, the enzymatic activity of TetX on Dox and ClTc was modeled by entering the known *in vitro K*_m _and *k*_cat _values of TetX for these compounds as fixed parameters [9]. The only free parameter in the fitting process was an arbitrary diffusion constant, *K *that accounted for the diffusion properties of Tc derivatives. Although parameters for the diffusion of Tc into *E. coli *have been experimentally determined [14,15], the value of *K *for our fitting could not be determined *a priori *because it is not known how the diffusional properties of Tc derivatives would change upon modification by TetX or how quickly the modified Tc derivatives might degrade within the cell. In fitting the data from each experiment, the data generated for TetX_D311A _was used as a reference to predict how much luminescence would be generated at a given effective concentration of drug in the absence of enzyme. It can be seen in Table 1 that we were able to fit the Dox and ClTc data effectively using the known kinetic parameters of TetX (R^2 ^values equaled 0.90 and 0.66 for Dox and ClTc, respectively). To fit the curves generated using Atc, for which *K*_m _and *k*_cat _values for TetX were not known, we allowed *K*_m _and *k*_cat _to also be free parameters. Notably, we were still able to obtain good fits to our data and the parameters returned were similar to those in the other fits. These data suggest that TetX acts on Atc with similar kinetic parameters as on Dox and ClTc. The ability to obtain fits to different experiments with consistent enzyme parameter values supports our conclusion that the behavior of this system is the result of the enzymatic activity of TetX against Tc derivatives. The use of the data generated from the pET_*tetXD*_-containing cells as the reference curve requires that the time taken after drug addition for the intracellular drug concentration to reach equilibrium in pET_*tetX*_-containing cells (*t*) is short enough to not affect luminescence accumulation. With the enzyme parameters from the above data fitting, we were also able to estimate *t *(see Methods for details). As shown in Table 1, *t *was less than 10 min in all experiments, which is much shorter than the time at which luminescence was measured (2-4 hours).

**Table 1:** Fitting results of cell based TetX activity experiments^a^

Enzyme	Experiment	Substrate	**R**^**2**^	***K***_***m ***_**(μM)**	***k***_***cat ***_**(s**^**-1**^**)**	[Enzyme] (μM)	***K *(10**^**-3**^**)**	*t *(s)
TetX	Figure 3A	Atc	0.90	***130***	***0.7***	15	***3.5***	***37***
TetX	Figure 3B	Atc	0.96	***160***	***0.4***	15	***12.2***	***41***
TetX	Figure 3C	Dox	0.90	83	0.6	15	***6.9***	***27***
TetX	Figure 3D	ClTc	0.66	110	0.3	15	***2.9***	***70***

^a ^Fixed parameters were shown in regular fonts and values returned from fitting were shown in bold italic fonts

## Conclusions

In the work described here we have developed a sensitive luminescence-based *in vivo *assay to detect enzymatic activity against tetracyclines. Our assay system is based on the ability of these compounds to induce TetR regulated transcription and the diminution of this induction that occurs when a Tc modifying enzyme activity is present within the cell. An important aspect of this system is that it not only detects enzyme activity, but also allows quantitation of this activity. The only requirement is that the Tc derivative is modified in a way that lowers its affinity for TetR. Of course, our system will fail to detect an enzyme activity if the modification produced does not change the affinity of the Tc derivative for TetR. However, due to the sensitivity of our assay system and the use of titrations to detect activity, even a small change in affinity would be detected. In addition, there are a number of TetR mutants with varying reactivities towards different Tc derivatives [16,17] that could be utilized in our assay system to maximize the number of Tc modifications that could be detected. Finally, there are other TetR-like repressors that are induced by Tc derivatives and at least one of these, TtgR of *Pseudomonas putida *[18], binds to Tc by a completely different mechanism compared to TetR. By using these different repressors, a wide range of modifications of tetracyclines would likely be detectable. Since there is great interest in the identification of enzymes that may modify tetracyclines to produce more potent antibiotics [19], our assay could be useful as a rapid screen to determine the level of activity of a given enzyme against a range of tetracyclines. Because of the wide range of ligands bound by TFRs [6], the assay system described here could be adapted to test for enzymatic activities against a huge variety of antibiotics and other small molecules. Supporting the general utility of our system, we have constructed reporters analogous to pYR_*tetOR *_for 22 diverse TFRs, and all of these TFRs are able to repress transcription of the *lux *operon when expressed in *E. coli *(data not shown). A reporter similar to the one described here was used to discover new ligands for the ActR repressor of *S. coelicolor *[20,21]. Future studies will determine whether enzyme activities for a variety of small molecules will be detectable using these systems. It should be noted that the same principles used to design this TetR-based system could be used for any of the other families of transcriptional regulators that are induced by small molecules.

In general, our TetR-based system or other systems designed in a similar manner present many advantages for investigation of enzymes with activities against small molecules. First, we are able to detect enzyme activity using only nanogram quantities of compound. This feature could be critical in screening for activities against compounds that are available only in small quantities as in the case of compound libraries used for high-throughput screening. The ability to modulate the expression level of the repressor in our system by addition of varying concentrations of arabinose allows the intracellular repressor concentration to be adjusted to a level at which there is just enough present to repress transcription. Consequently, addition of only a small amount of inducer is required for derepression of the system. A second advantage of our system is that it provides the potential to investigate enzymatic activities without having to purify the enzymes or know their co-factor requirements. Our assay system would be equally capable of measuring the activity of difficult to purify membrane proteins, such as drug efflux pumps. TetR-based reporter systems have been shown to function in a wide range of cell types including mammalian cells [22-24], thus, enzymatic assays operating by the same principle as ours could be adapted to many cell types. A final advantage of our system is that it could be used to screen for enzymes with activity against a given compound of interest. For this purpose, a library of plasmids expressing candidate enzymes would be transformed into a strain containing a pYR-derivative that responded to the compound of interest. Individual colonies could then be screened in a 96-well format for reduced luminescence resulting from expression of an enzyme that modified the TFR-binding molecule. In this way, it will be possible to systematically identify novel enzymes with activities against many important small molecules.

## Methods

### Bacterial strains, plasmid constructs and culture conditions

Bacterial strains and plasmids used in this work are shown in Additional file1: Table S1. *E. coli *cells were grown at 37°C in Luria broth (LB) or LB agar medium containing the following antibiotics when necessary: kanamycin (50 μg/mL), ampicilin (100 μg/mL) and streptomycin (50 μg/mL). Protein expression for purification and *E. coli *drug susceptibility assays were carried out in *E. coli *BL21*(DE3). *In vivo *repression and induction assays were performed using *E. coli *Top10 (Invitrogen) because it is an arabinose metabolism deficient strain and produces more luminescence than BL21*(DE3). A T7 polymerase bearing *E. coli *Top10 (DE3) strain was constructed for the *in vivo *enzymatic activity assay using the Lambda DE3 Lysogenization Kit from Novagen.

### DNA manipulation procedures

Standard procedures were employed for all DNA manipulation and molecular cloning [25]. The oligonucleotides and primers used in this study were synthesized from the ACGT Corporation (Toronto) and listed in Additional file1: Table S2. PCR reactions were carried out using Vent DNA polymerase (New England Biolabs). The QuikChange site-directed mutagenesis protocol (Stratagene) was employed to create point mutations of TetX.

### Construction of the pYR plasmids and protein expression vectors

The pCS26-Pac plasmid [11], which contains the *luxCDABE *operon on a low copy number pSC101-derived vector [26], was modified by inserting a double stranded oligonucleotide (oligo YLuxupdateF annealed to oligo YLuxupdateR) upstream of *luxCDABE *operon in between *Xho*I and *Bam*HI sites. The araBAD promoter and the *araC *gene from the pBAD vector (Invitrogen), were amplified using primers SR204 and SR205 and then ligated into the *Eco*RI and *Kpn*I restriction sites introduced as described above, creating the pYR plasmid (~13 kbp). A synthetic promoter consisting of the TetR operator and promoter *tetO *from the Tn*10 tetA *promoter region was prepared by annealing YZ201 and YZ202 oligonucleotides. This promoter was then introduced into pYR between *Kpn*I and *Pml*I upstream of the *luxCDABE *cluster to give pYR_*tetO*_. The *tetR *gene was amplified from pET_*tetR *_[27] using primer YZ203 and YZ204 and cloned into pYR_*tetO *_between *Xho*I and *Eco*RI, downstream of the araBAD promoter, to give pYR_*tetOR*_. Expression vector pET_*tetX *_was prepared by introducing the PCR amplified *tetX *gene using primer pair YZ241-2 YZ242 into pET21 between *Eco*RI and *Xho*I sites.

### Luminescence assays

For the repression and induction assays, isolated *E. coli *colonies were used to inoculate 2 mL cultures, which were grown overnight. 10 μL of overnight culture was added into 2 mL of LB media in the presence of varying concentrations of arabinose and drugs. These cultures were grown for 12 to 16 h before measurement of luminescence using a BMG Fluostar OPTIMA luminometer. In the enzyme assays, we inoculated 4 μL overnight cultures into 200 μL fresh pH 7 buffered LB media containing 0.04% glycerol, then grew for 2-4 h until early log phase and added varying concentrations of drugs. Luminescence and optical density were measured every 15 min using a TECAN Infinite M200 luminometer. Luminescence from similar early stationary phase cells (around 2-4 h after drug induction) was used to calculate the induction ratio. Multiple replicates (N > = 2) were performed and the 95% confidence intervals which are 1.96 times standard errors were displayed as error bars.

### Enzyme Kinetics Data Fitting for Enzyme-Containing Cells

In the presence of TetX, the effective cytoplasmic inducer concentration, [*I*_*eff*_] was expected to reach steady state when the enzyme catalytic rate equalled the diffusion rate. The enzyme catalytic rate was expressed using the Michaelis-Menten equation:

(1) $$v_{t}=k_{cat}\cdot [E]\cdot \frac{\left[I_{eff}\right]}{K_{m}+[I_{eff}]}$$

where *v*_*t *_is the rate of reaction at time point *t *after inducer addition with an intracellular inducer concentration of [*I*_*eff*_] at that time point, *k*_*cat *_is the catalytic rate constant, *K*_*m *_is the Michaelis constant and [*E*] is the enzyme concentration, which was assumed to be constant. The concentration of enzyme was estimated through analysis of Western Blots (see below). To determine [*I*_*eff*_] as a function of the extracellular inducer concentration ([*I*_*out*_]), the diffusion process across the cell membranes was modeled. Diffusion is normally described by the Fick's first law of diffusion:

(2) $$v_{d}=P_{0}\cdot A_{0}\cdot [I_{out}-I_{eff}]$$

where *v*_*d *_is the diffusion rate, *P*_*0 *_is the permeability coefficient through diffusion barrier, and *A*_*0 *_is the barrier surface area. Although *P*_*0 *_and *A*_*0 *_are known for Tc [28], to simplify the model and generalize it to situations where *P*_*0 *_and *A*_*0 *_might not be known for a given compound, we replaced these parameters with an arbitrary diffusion constant *K*. The diffusion process was then expressed by:

(3) $$v_{d}=K\cdot [I_{out}-I_{eff}]$$

*K *accounts for not only *P*_*0 *_and *A*_*0*_, but also the diffusion-effective concentration difference across the cell membranes. Since enzymes may only make subtle changes on the inducer, it is impossible to predict how these changes would affect diffusion properties. In addition, some enzymatic modifications will lead to inducer degradation. In the steady state, *v*_*t *_equals to *v*_*d*_, therefore the inducer concentration [*I*_*eff*_] could be determined by solving:

(4) $$\begin{array}{l} k_{cat}\cdot [E]\cdot \frac{\left[I_{eff}\right]}{K_{m}+[I_{eff}]}=K\cdot [I_{out}-I_{eff}]\Rightarrow \\ {}[I_{eff}]=\frac{-(k_{cat}\cdot [E]/K-[I_{out}]+K_{m})+\sqrt{{\left(k_{cat}\cdot [E]/K-[I_{out}]+K_{m}\right)}^{2}+4\cdot K_{m}\cdot [I_{out}]}}{2} \end{array}$$

Thus, the steady state [*I*_*eff*_] can be described as a function of three parameters: *K*, *K*_*m *_and *k*_*cat*_. The relationship between [*I*_*eff*_] and the luminescence induction ratio (*L*) was determined from curves generated for pET_*tetXD*_-containing cells, which were assumed to contain no active TetX. In this situation, the steady state [*I*_*eff*_] and [*I*_*out*_] were assumed to be equivalent. While this may not be strictly true in all conditions (in fact, the intracellular concentration of Tc has actually been found to be 4-fold higher than the extracellular concentration under some conditions [15]), the success of our fitting procedure requires only that [*I*_*eff*_] and [*I*_*out*_] are related by a constant value over the range of inducer concentrations used. Any deviation from equivalence of [*I*_*eff*_] and [*I*_*out*_] is accounted for in the *K *value described above, which is a free parameter in our fitting scheme. The curves of *L *versus [*I*_*out*_] for the pET_*tetXD*_-containing cells were fit to a hyperbolic equation (in the presence of 0.0002% arabinose) or a sigmoidal model (in the presence of 0.02% arabinose) as shown:

(5) $$L=\frac{a\cdot [I_{out}]}{b+[I_{out}]}+c\cdot [I_{out}]$$

(6) $$L=L_{0}+\frac{a\cdot {\left[I_{out}\right]}^{b}}{c^{b}+\cdot {\left[I_{out}\right]}^{b}}$$

where *a, b, c *and *L*_*0*_, are arbitrary parameters giving the completed standard curves. Although these equations have no physical relevance to the functioning of the system, they accurately captured the relationship between *L *and [*I*_*out*_] under these conditions (R^2 ^> 0.9 in every fitting). Finally the *L *versus [*I*_*out*_] curves for pET_*tetX*_-containing cells were fitted to the following equations using the information derived from the fits of the pET_*tetXD*_-containing cells.

Under 0.0002% arabinose:

(7) $$L=\{\begin{array}{cc} \frac{a\cdot [I_{out}]}{\left(b+[I_{out}]\right)}+c\cdot [I_{out}], & \left[I_{out}]\leq [I_{s}\right]\\ \frac{a\cdot [I_{eff}]}{\left(b+[I_{eff}]\right)}+c\cdot [I_{eff}], & \left[I_{out}]>[I_{s}\right] \end{array}$$

Under 0.02% arabinose:

(8) $$L=\{\begin{array}{cc} L_{0}+\frac{a\cdot {\left[I_{out}\right]}^{b}}{\left(c^{b}+{\left[I_{out}\right]}^{b}\right)}, & \left[I_{out}]\leq [I_{s}\right]\\ L_{0}+\frac{a\cdot {\left[I_{eff}\right]}^{b}}{\left(c^{b}+{\left[I_{eff}\right]}^{b}\right)}, & \left[I_{out}]>[I_{s}\right] \end{array}$$

The equations above also took into account the competition for drugs from TetR. Since TetR binds Tc derivatives much more tightly than TetX, TetX will only induce when TetR is saturated. The TetR saturating inducer concentration [*I*_*s*_] was set to be the inducer concentration corresponding to the onset of luminescence induction. For fitting curves to data derived from pET_*tetX*_-containing cells in response to Dox and ClTc, only the *K *value was left as free parameters and published *in vitro K*_*m *_and *k*_*cat *_values were used.

The steady state [*I*_*eff*_] can also be expressed as a function of the equilibration time *t*:

(9) $$[I_{eff}]=\int_{0}^{t}(v_{d}-v_{t})\cdot tdt$$

where *v*_*d *_was determined from equation (3) and *v*_*t *_was determined from equation (1). With *K*, *K*_*m *_and *k*_*cat *_determined in previous steps, *t *became the only free parameter and can be returned by data fitting using equation (7) or (8).

### Estimation of Intracellular Enzyme Concentrations

To estimate *in vivo *enzyme concentrations, TetX-bearing cells were harvested at the time when drugs were added to induce luminescence. Approximately 1 μL cell pellet was lysed and loaded on a SDS-PAGE and subjected to a Western blot using anti-6xHis antibody. A series of purified 6xHis tagged TetR was loaded on the same gel as concentration standards (Figure 3E). TetX was estimated to be 15 μM.

### Protein expression and purification of repressors and enzymes

Proteins were expressed as C-terminal hexahistidine fusions in *E. coli *strain BL21*(DE3) using plasmids derived from pET21d (Novagen). TetR was purified as previously described [27]. For TetX and mutants, cells bearing pET_*tetX *_were grown at 30°C to an OD_600 _of 0.8, induced with 1 mM IPTG, and the grown overnight at 20°C. Cells were harvested and lysed in 6 M GuHCl. Protein was purified using nickel affinity chromatography by the denatured protein procedure (Qiagen). TetX was refolded by dialyzing into 10 mM Tris-HCl (pH 8.0), 50 mM NaCl, 0.1 mM EDTA, 1 mM DTT and 2% glycerol. These proteins were further purified through a hi-load sephadex-75 gel filtration column (Pharmacia) using a Pharmacia LKB FPLC. All *in vitro *protein assays were performed in the dialysis buffer described above.

### In vitro enzymatic assays

Fluorescence assays were performed using an Aviv ATF 105 spectrofluorometer in a 1 cm path length cuvette. Complex solutions with the indicated concentration of ingredients were excited at 340 nm and emission between 350 and 600 nm was recorded over time, averaging for 2 seconds at each wavelength.

## Abbreviations

TetR: tetracycline repressor; TFR: TetR family of transcriptional regulators; Tc: Tetracycline; Atc: Anhydrotetracycline; Dox: doxycycline; ClTc: chlorotetracycline; MIC: minimum inhibitory concentration.

## Authors' contributions

ZY, SER and LC performed research; ZY analyzed data; ZY, SER, JRN and ARD designed research; and ZY and ARD wrote the manuscript. All authors read and approved the final document.

## Supplementary Material

Additional file 1 **Additional figures and tables for *Characterization of tetracycline modifying enzymes using a sensitive in vivo reporter system ***This file contains additional figures and tables of the main manuscript Click here for file
