# Supplementary material for: Characterization of tetracycline modifying enzymes using a sensitive in vivo reporter system
Source: BMC Biochem. 2010 Sep 11;11:34. doi: 10.1186/1471-2091-11-34 (PMC2949611; doi:10.1186/1471-2091-11-34)
Supplement: Additional file 1 — Additional figures and tables for Characterization of tetracycline modifying enzymes using a sensitive in vivo reporter system This file contains additional figures and tables of the main manuscript [file 1471-2091-11-34-S1.PDF]

Additional File 1

Additional figures and tables for

Characterization of tetracycline modifying enzymes using a sensitive *in vivo* reporter system

Zhou Yu[1], Sean E. Reichheld[2], Leslie Cuthbertson[3], Justin R. Nodwell[3] and Alan R. Davidson[1,2]*

[1] Department of Molecular Genetics, [2] Department of Biochemistry, University of Toronto, 1 King's College Circle, Toronto, ON, M5S 1A8, Canada

[3] DeGroote Institute for Infectious Diseases Research, Department of Biochemistry and Biomedical Sciences, McMaster University, 1200 Main Street W, Hamilton, ON, L8N 3Z5, Canada

[*] Corresponding Author: Dr. Alan R. Davidson

Figure S1: Structure and sequence of TetX-like enzymes. A) Structure of a TetX-like enzyme, the hydroxybenzoate hydroxylase Phbh of *Pseudomonas aeruginosa* (PDB 1K0I). Proteins of this family consist of two domains, a substrate binding domain (gray ribbons) and a FAD binding domain (green ribbons). FAD is shown in yellow sticks and the Aspartic acid that was mutated to Alanine is shown in red sticks. The red dotted lines illustrate hydrogen bond interactions. **B)** Sequence alignment of TetX-like enzymes, colored using the ClustalX scheme. Positions that are within 5 Å of FAD in the Phbh-FAD complex shown in A are indicated by black boxes. These residues are conserved across the whole family. The conserved Aspartic acid used in creating inactive enzymes is indicated by an arrow.

Figure S2: *In vitro* fluorescence-based assay of TetX activity. When Tc is excited with light at 340 nm, it emits fluorescence at 520 nm. We observed that subtle modifications of Tc can significantly alter its fluorescent properties. For example, Atc differs from Tc at only one hydroxyl group, yet it emits a much lower level of fluorescence with a peak at 540 nm. Thus, we reasoned that covalent modification of Tc by TetX would result in a change in its fluorescent properties. Consistent with our expectation, when purified TetX in the presence of its required co-factors, FAD and NADPH [1] was added to Tc, a significant reduction in fluorescence at wavelengths between 400 and 600 nm was observed over a time period of approximately 1 hour **(A)**. This change in fluorescence is attributable to a reduction in the concentration of Tc, which emits with a peak at 520 nm, and to a reduction in the concentration of NADPH, which emits with a peak at 460 nm. Assays performed in the absence of FAD or NADPH displayed no changes in fluorescence over time (data not shown). Importantly, a reaction including the TetX D311A mutant also displayed no significant change in fluorescence over time **(B)**, implying that substitution of Asp 311 indeed abrogated the enzymatic activity of TetX. This result confirmed our prediction from structural and sequence analysis that this residue is crucial for FAD-binding (Figure S1), and also justified our use of the D311A mutant as the negative control for the *in vivo* assays. The fluorescence emission scans were resulted from excitation at 340 nm in the presence of 1 mM $MgCl_2$, 5 μM FAD, 5 μM NADPH and 5 μM Tc. Scans were performed every 6 minutes over 54 minutes.

Fluorescence assays were performed using an Aviv ATF 105 spectrofluorometer in a 1 cm path length cuvette.

Table S1: Bacterial strains and plasmids used in this work

Strain/plasmid	Genotype/resistance marker	Background	Source/Reference
Strains			
E. coli			
BL21*(DE3)	F^- *dcm ompT hsdS*(rB^- mB^-) *gal met* λ(DE3)		Novagen
Top10	F^- *mcrA Δ(mrr-hsdRMS-mcrBC) φ80lacZΔM15 ΔlacX74 nupG recA1 araD139 Δ(ara-leu)*7697 *galE15 galK16 rpsL*(Str^R) *endA1* $λ^-$		Invitrogen
Top10(DE3)	F^- *mcrA Δ(mrr-hsdRMS-mcrBC) φ80lacZΔM15 ΔlacX74 nupG recA1 araD139 Δ(ara-leu)*7697 *galE15 galK16 rpsL*(Str^R) *endA1* λ(DE3)		This work
S. coelicolor			
M145	*S. coelicolor* prototroph, $SCP1^-$ $SCP2^-$		Dr. Nodwell, J
Plasmids			
pET21	IPTG inducible expression vector based on T7 promoter (Amp^R)	pBR322	Novagen
pCS26-Pac	Promoterless *luxCDABE* reporter (Kan^R)	pSC101	[2]
pBAD	Plasmid bearing the pBAD promoter and AraC repressor AraBAD (Amp^R)	pBR322	Invitrogen
pYR	AraBAD bearing pCS26-Pac (Kan^R)	pCS26-Pac	This work
pYR_{tetO}	Reporter *luxCDABE* located downstream of a promoter bearing TetR operator *tetO* (Kan^R)	pYR	This work
pYR_{tetOR}	TetR located downstream of AraBAD (Kan^R)	pYR_{tetO}	This work
pET_{TetX}	His_6-TetX fusion expression vector (Amp^R)	pET21	This work
pET_{tetR}	TetR(B) expression vector (Amp^R)	pET21	[3]
$pET28_{tetX}$	TetX bearing vector (Kan^R)	pET28	Dr. Wright, G

Table S2: Oligonucleotides and primers used in this study

Name	Sequence (5'-3')	Purpose
YLuxupdateF	`tcgaggctagcgaattcggtacccacgtggtcgacgggcccg`	Forward oligonucleotide for a multiple restriction site to be inserted into pCS26-Pac
YLuxupdateR	`gatccgggcccgtcgaccacgtgggtaccgaattcgctagcc`	Reverse oligonucleotide for a multiple restriction site to be inserted into pCS26-Pac
SR204	`ccacgtggtaccacagacaagctgtgaccgtctc`	Forward primer for amplifying *araBAD* to be inserted into pCS26-Pac using pBAD as template
SR205	`gctagcgaattccatggttaattcctcctgttagc`	Reverse primer for amplifying *araBAD* to be inserted into pCS26-Pac using pBAD as template
YZ201	`cgttgacactctatcattgatagagttattttacca`	Forward oligonucleotide for preparing the *tetO*
YZ202	`tggtaaaataactctatcaatgatagagtgtcaacggtac`	Reverse oligonucleotide for preparing the *tetO*
YZ203	`ggaagaattcgctagattagataaaagtaaagtgattaac`	Forward primer for amplifying *tetR* using pET_{tetR} as template
YZ204	`gaaactcgagttaagacccactttcacatttaag`	Reverse primer for amplifying *tetR* using pET_{tetR} as template
YZ241-2	`ggaagaattcatgaatttacttagtgataagaac`	Forward primer for amplifying *tetX* using $pET28_{tetX}$ as template
YZ242	`gaaactcgagtacatttaacaattgctgaaacgt`	Reverse primer for amplifying *tetX* using $pET28_{tetX}$ as template

References

1. Yang W, Moore IF, Koteva KP, Bareich DC, Hughes DW, Wright GD: **TetX is a flavin-dependent monooxygenase conferring resistance to tetracycline antibiotics**. *J Biol Chem* 2004, **279**(50):52346-52352.
2. Bjarnason J, Southward CM, Surette MG: **Genomic profiling of iron-responsive genes in Salmonella enterica serovar typhimurium by high-throughput screening of a random promoter library**. *J Bacteriol* 2003, **185**(16):4973-4982.
3. Reichheld SE, Davidson AR: **Two-way interdomain signal transduction in tetracycline repressor**. *J Mol Biol* 2006, **361**(2):382-389.

Figure S1

A

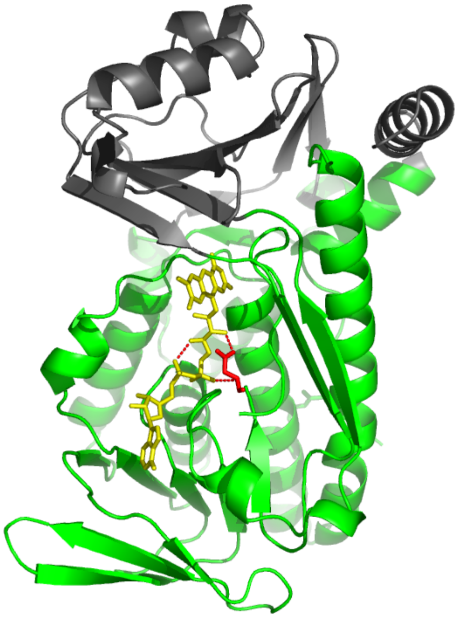

B

```
TetX             1 MTMRIDTDKQMNLLSDKNVAIIGGGPVGLTMAKLLQQNGIDVSVYERDNDREARIFGGTLDLHKGSGQEAMKKAGLLQTYYDLALP-MGVNIADKKG-----NILS
Sco0252          1 -------MAQRSGTTSLRVLVAGGGIAGHALAYWLARGGH--RVTVRERYPDLRATGAQIDLR-GQGIEAVRRMGLLDTVRGRLVDEAGVAFLDARGRPRATLMAN
OxyE             1 ---MTGHPRPPADGAHTDVCVVGGGPAGLTLALLMLRSGAR--VTLVERSRSLDRAYRGEILQ-PGGQALLDALGVLEGARRRGCHEHDGFRLEERG---RTLING
PhzS_PDB:2RGJ    1 ------------MSEPIDILIAGAGIGGLSCALALHQAGIG-KVTLLESSSEIRPLGVGINIQ-PAAVEALAELGLGPALAATAIPTHELRYIDQSG---ATVWSE
PHBH_PDB:1K0I    1 --------------MKTQVAIIGAGPSGLLLGQLLHKAGID-NVILERQTPDYVLGRIRAGVLEQGMVDLLREAGVDRRMARDGLVHEGVEIAFAGQ------RRR

TetX           101 TKNVKPENRFDNPEINRNDLRAILLNSLEN----DTVIWDRKLVMLEPGKKKWTLTFENKPSE----TADLVILANGGMSKVRKFVTDT--EVEETGTFNIQADIH
Sco0252         97 TSGRGRQSLTSEYEIMRGDLVRILHEATGN---DVEYVYGTSVDGFDQDEHEVVAHCSDGSSE----TYDLLVAADGQGSRLRRAILPD------GTDPYWRVGIH
OxyE            98 DYRRLPGPYNCLLSLPQQHLLTDLLERCRAHP-RFTCLTGTKVNGLVEEGGVVRGVVCGGGADGLVVRADCVVGADGRYSTVR----------KLAGIPYDRIELF
PhzS_PDB:2RGJ   90 PRGVEAGNAYPQYSIHRGELQMILLAAVRERLGQQAVRTGLGVERIEERDGRVLIGARDGHGKPQALGADVLVGADGIHSAVRAHLHPDQRPLSHGGITMWRGVTE
PHBH_PDB:1K0I   86 IDLKRLSGGKTVTVYGQTEVTRDLMEAREACG--ATTVYQAAEVRLHDLQGERPYVTFERDGERLRLDCDYIAGCDGFHGISR------------QSIPAERLKVF

TetX           197 QPEINCPGFFQLCNG---NRLMASHQGNLLFANPNNNGALHFGISFKTPDEWKNQTQVDFQNRNSVVDFLLKEFSDWDERYKELIHTTLSFVGLATRIFPLEKPWK
Sco0252        190 MAYWFVPRIASDSNV---RDTCMVPGGRQIMRRSHNPTETQVYFVLREDSAQASAIHREPVEKQQEFWASRFRDAGWQTERFVEG---MRTSPFFYSQEIVQVRTG
OxyE           193 DQDVLWCKLTAPATR---TVRIFRAGGNPVLAYTSFPDCVQLGWTLP-HKGYQALAERGFAHVKERIRAAVPEYADTVDQQLNSFKDVSLLDVFAG-------SAR
PhzS_PDB:2RGJ  196 FDRFLDGKTMIVAND---EHWSRLVAYPISARHAAEGKSLVNWVCMVPSAAVGQLDNEADWNRDGRLEDVLPFFADWDLGWFDIRDLLTRNQLILQYPMVDRDPLP
PHBH_PDB:1K0I  178 ERVYPFGWLGLLADTPPVSHELIYANHPRGFALCSQRSATRSQYYVQVPLSEKVEDWSDERFWTELKARLPSEVAEKLVTGPSLEKSIAPLRSFVVEP--------

TetX           300 SKRPLPITMIGDAAHLMPPFAGQGVNSGLVDALILSDNLADG-------KFNSIEEAVKNYEQQMFMYGKEAQEE----STQNEIEMFKPDFTFQQLLNV-----
Sco0252        290 TWSRGRVVLAGDAAHCASPYSGMGTSGGLVGAHVLAGEINRRP-DDLPTALAHYDTTLRPFTDEIQGEVNPRLLKLAMPMTQRAIDAFLTVTGMATRLRVPDLAT
OxyE           288 RWARDGLLLIGDSAHTHSPIGAQGINLAIQDAVAAHPVLCEGL-RRRDLSERFLDAVAARRRPETERATRVQVMQSRMMLSTGRVSAAVRPKAAMLVSRTPAYRS
PhzS_PDB:2RGJ  299 HWGRGRITLLGDAAHLMYPMGANGASQAILDGIELAAALARN--ADVAAALREYEEARRPTANKIILANREREKEEWAAASRPKTEKSAALEAITGSYRNQVERP
PHBH_PDB:1K0I  276 -MQHGRLFLAGDAAHIVPPTGAKGLNLAASDVSTLYRLLLKAYREGRGELLERYSAICLRRIWKAERFSWWMTSVLHRFPDTDAFSQRIQQTELEYYLGSEAGLA
```

Figure S2

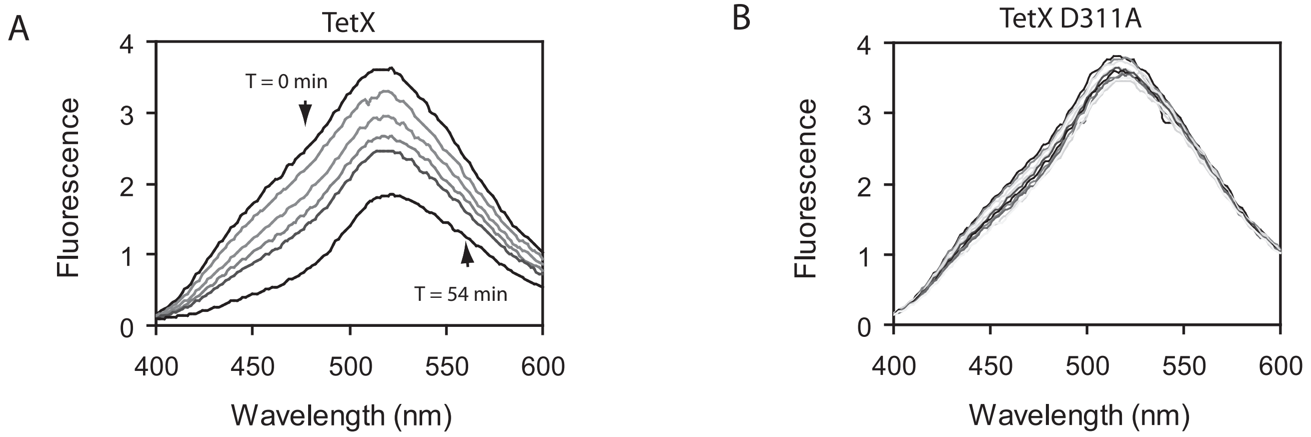
A
TetX
T = 0 min
T = 54 min
Fluorescence
Wavelength (nm)
0
1
2
3
4
400
450
500
550
600
B
TetX D311A
Fluorescence
Wavelength (nm)
0
1
2
3
4
400
450
500
550
600
